# Intestinal Development in Wuzhishan Pigs at Different Growth Phases: Insights from Microbiome and Metabolomics

**DOI:** 10.3390/ani16060976

**Published:** 2026-03-20

**Authors:** Jing Fan, Xinyu Xue, Haojie Zhang, Feng Wang, Zhe Chao, Limin Wei, Hailong Liu, Yuwei Ren, Ruiping Sun

**Affiliations:** 1 Key Laboratory of Tropical Animal Breeding and Epidemic Disease Research, Institute of Animal Husbandry and Veterinary Research, Hainan Academy of Agricultural Sciences, Haikou 571100, China; fj13359819865@163.com (J.F.); xxy2542999712@163.com (X.X.); wfeng73cn@sina.com (F.W.); chaozhe.cn@126.com (Z.C.); liminedu@126.com (L.W.); liuhailong423@126.com (H.L.); renyuwei@hnaas.org.cn (Y.R.); 2 National Engineering Research Center for Breeding Swine Industry, Chinese National Centre of Pig Breeding Technology, ALLTECH-SCAU Animal Nutrition Control Research Alliance, College of Animal Science, South China Agricultural University, Guangzhou 510642, China; 3 College of Animal Science and Technology, Guangxi University, Nanning 530005, China; zhanghj089@126.com

**Keywords:** intestinal microbiota, metabolites, intestinal morphology, developmental phases, Wuzhishan pig

## Abstract

Wuzhishan pigs are a typical Chinese indigenous miniature pig breed, with thin skin and high amino acid content in muscle; slow weight gain and long feeding phases limit their value. As the primary digestive and absorptive organ, the intestine is crucial for growth, yet current studies on its development are limited. This study aimed to investigate intestinal physiological differences in Wuzhishan pigs across four phases (pre-weaning: 7, 14 days; weaning: 35, 38, 45 days; fattening: 70, 100 days; maturity: 180, 240 days) by evaluating intestinal morphology, digestive enzyme activity, gut microbiota diversity via 16S rRNA gene sequencing, and metabolite characteristics via metabolomic analysis. Results showed poor intestinal morphology and enzyme activity during weaning, significant ileal and colonic microbial diversity differences across phases, increased beneficial bacteria with age, and enriched opportunistic pathogens (*Streptococcus*, *Romboutsia*, *Terrisporobacter*) during weaning; weaning also had lower lipid metabolites, correlated with decreased *Fusobacterium*, *Lactobacillus*, and *Muribaculaceae*. Fattening enhanced amino acid metabolism, with increased *Lactobacillus* correlated with higher amino acids and muscle-related metabolites, while maturity increased immune-related metabolites (e.g., pyridoxine) in the vitamin B6 pathway. These results explain delayed rapid weight gain in Wuzhishan pigs and provide a theoretical basis for maintaining intestinal stability and production performance.

## Simple Summary

Wuzhishan pigs are an important indigenous Chinese breed with relatively slow growth. This study investigated intestinal development, gut microbiota, and metabolites across four growth stages. Intestinal function was immature at weaning, with a higher abundance of potential opportunistic pathogens and lower lipid metabolites. During fattening, amino acid metabolism was significantly enhanced, which was associated with increased abundance of Lactobacillus. In mature pigs, immune-related metabolites were elevated and enriched in vitamin B6 metabolism. These sequential changes in gut microbes and metabolites provide a basis for clarifying the mechanism underlying accelerated weight gain after the fattening stage. This study reveals intestinal developmental patterns in Wuzhishan pigs and supports improving their growth efficiency.

## 1. Introduction

Local pig breeds are formed through long-term natural and artificial selection in specific regions and are adapted to the local environmental conditions. These pig breeds have high economic value, strong ecological adaptability, and rich genetic diversity. The Wuzhishan pig is one of the few miniature local pig breeds in China and is native to Hainan Island. It exhibits strong adaptability and inbreeding tolerance, and is characterized by abundant fatty acids and amino acids in meat [1]. Owing to these characteristics, Wuzhishan pigs have high research and development value and have attracted the attention of researchers. However, as a miniature pig breed, Wuzhishan pigs show slow growth, which limits their production and application. At present, the characteristics of intestinal development, gut microbiota, and metabolites at different growth phases remain unclear in this breed. Therefore, exploring the physiological characteristics of intestinal development at different phases is necessary to improve the growth performance of Wuzhishan pigs.

The intestine is an important digestive and immune organ, and maintaining its health is crucial for growth and disease prevention. From birth to adulthood, the intestinal system of pigs undergoes dynamic changes, with significant differences in the digestive enzymes, morphology, and microbial abundance at different phases of growth and development. For example, intestinal amylase, trypsin, and lactase activities are higher in suckling piglets, whereas lactase activity decreases after weaning [2,3]. The villus height (VH) in finishing pigs is the highest, while the crypt depth (CD) is the deepest. Furthermore, villi atrophy occurs during the weaning phase, and the division of crypt cells accelerates, resulting in significant changes in intestinal morphology [4]; The dominant bacterial genera in the intestines of pigs during the pre-weaning phase are *Lactobacillus* and *Bacteroide* [5]. While the abundance of *Lactobacillus* decreases during the weaning phase, that of pathogenic bacteria such as *Escherichia coli* and *Salmonella* increases [6]. Following the weaning phase, *Bifidobacterium* gradually becomes the dominant genus [7]. The difference in growth and developmental phases is a decisive factor that affects the succession of intestinal microbiota in pigs [8]. Therefore, exploring the intestinal physiological differences in Wuzhishan pigs at different developmental phases is crucial for improving pig production performance and health.

Trillions of microorganisms live in the gastrointestinal tracts of pigs and are collectively referred to as the intestinal microbiota [9]. This large and complex microbiota is involved in a series of biological processes specific to pigs that are closely related to host health, including immune regulation, nutrient metabolism, and growth and development, as reported in porcine studies [10]. Li et al. found that *Lactobacillus* in piglets plays an important role in the intestinal immune regulation of piglets by promoting the expression of cyclooxygenase-2 and inducible nitric oxide synthase in the porcine intestine [11]. In pigs, intestinal microorganisms in pigs are important participants in nutrient metabolism, as they decompose digestive tract nutrients and provide hosts with beneficial raw materials to promote growth and development. Lipid metabolism is closely related to microorganisms such as *Clostridium*, *Bacteroides*, and *Lactobacillus* [12]. Fibrous substances can be degraded by intestinal bacteria such as *Bacteroides*, *Ruminococcus*, and *Roseburia* to produce short-chain fatty acids, which provide energy to intestinal epithelial cells and extraintestinal tissues [13]. In recent years, metabolites and functional mechanisms derived from intestinal microorganisms have been gradually revealed. Metabolites serve as signaling molecules and substrates for metabolic reactions in the body, affecting the physiological and pathological processes of the host. Studies have suggested that pyridoxine produced by *Bacteroides* and *Fusobacteria* may contribute to vitamin supplementation and immune modulation in the host [14]. In addition, evidence indicates that secondary bile acids associated with *Lactobacillus* and *Bifidobacterium* may be involved in the absorption of dietary fats and fat-soluble vitamins, thereby supporting lipid absorption [15]. Therefore, using high-throughput sequencing and multi-omics techniques to understand the interaction between intestinal microbiota and host metabolism is helpful for accurately regulating the intestinal microbiota, thereby promoting pig health and production efficiency.

Wuzhishan pigs are a miniature pig breed with strong stress resistance and good meat quality. However, the physiological characteristics, dynamic changes in intestinal microbiota, and metabolite characteristics at different phases of intestinal development remain unclear. Therefore, this study explored the differences in intestinal morphology, digestive enzyme activities, intestinal microbiota, and metabolites of Wuzhishan pigs at different developmental phases, aiming to reveal the following: (1) the physiological differences in the intestinal microbiota of Wuzhishan pigs at different developmental phases; (2) the composition and characteristics of the intestinal microbiome and metabolome of pigs at various growth and developmental phases; (3) the possible correlation between intestinal microbiota and host metabolites, so as to provide a theoretical reference for guiding the nutritional regulation, improving intestinal stability, and enhancing production performance of Wuzhishan pigs.

## 2. Materials and Methods

### 2.1. Ethics Statement

All the animal experiments were approved by the Animal Ethics Committee of the Institute of Animal Husbandry and Veterinary Medicine, Hainan Academy of Agricultural Sciences (Ethics Code Permit: HNXMSY20230112).

### 2.2. Experiment Design and Animal Sampling

Wuzhishan pigs were selected at different growth phases: 12 at the pre-weaning phase (7 and 14 days old), 18 at the weaning phase (35, 38, and 45 days old), 12 at the fattening phase (70 and 100 days old), and 12 at the maturity phase (180 and 240 days old). All these Wuzhishan pigs were obtained from the National Germplasm Conservation Farm for Wuzhishan Pigs, Institute of Animal Husbandry and Veterinary Medicine, Hainan Academy of Agricultural Sciences, China. In addition, healthy pigs from the same batch, age, feeding method, and epidemic prevention system were selected for optimum results. The pig house was equipped with a natural ventilation system and fully slatted floor. Throughout the experiment, all pigs had free access to feed and water. Suckling piglets in the lactation phase were fed only sow milk, with no prestarter feed provided. Wuzhishan piglets were weaned at 35 days of age, and a gradual adaptation program was implemented to help them transition to solid feeding: specifically, from day 35 to day 41 postnatal, the piglets were fed a mixture of sow milk and the solid diet, with the proportion of solid feed gradually increased from 30% to 70%; from day 42 onwards, the piglets were fully fed the solid diet to ensure complete adaptation. Meanwhile, other pigs were fed a commercial solid diet purchased from Chia Tai Group (Bangkok, Thailand), and the nutritional composition values in Table 1, Table 2, Table 3 and Table 4 were determined by Guangzhou Kingzhi Testing Technology Co., Ltd. (Guangzhou, China) using standard Chinese national feed analysis methods (GB/T series), including crude protein (GB/T 6432-2018), crude fat (GB/T 6433-2025, modified from ISO 6492:1999), crude fiber (GB/T 6434-2022 modified from ISO 6865:2000), moisture (GB/T 6435-2014, modified from ISO 6496:1999), calcium (GB/T 6436-2018), total phosphorus (GB/T 6437-2018), crude ash (GB/T 6438-2025, modified from ISO 5984:2022), and amino acids (GB/T 18246-2019) [16,17,18,19,20,21,22,23,24,25,26,27]. The vitamin and trace element levels comply with the recommended standards by the NRC (2012) [28]. For sample collection, all pigs were weighed before slaughter. Prior to sampling, pigs were electrically stunned for immobilization and sacrificed by exsanguination according to standard humane slaughter procedures. After slaughter, the ileal and colonic contents were snap-frozen immediately in liquid nitrogen within 3 min and stored at −80 °C until analysis for bacterial community and metabolites. Intestinal segments (1.5 cm) were collected from the following standardized anatomical locations: Duodenum: 10 cm distal to the pylorus; Jejunum: 20 cm distal to the duodenal–jejunal flexure; Ileum: 15 cm proximal to the ileocecal junction; Cecum: the middle portion of the cecum; Colon: the middle portion of the ascending colon. The mucosa was scraped from these intestinal segments for intestinal enzyme activity measurement, and the fixed intestinal segments were used for histological examination.

### 2.3. Intestinal Morphology

Prepared using the standard paraffin embedding technique, transverse sections of duodenum, jejunum, and ileum samples were fixed with 4% paraformaldehyde, embedded in paraffin, sectioned (thickness, 5 μm), and then stained with haematoxylin and eosin for histological evaluation [29]. Microphotographs were captured using a Leica DM2000 light microscope (Leica, Wetzlar, Germany) at a magnification of 10×. Caseviewer Software (version 2.7, 3DHISTECH Ltd., Budapest, Hungary) was used to measure the VH, villus width (VW), and CD of the intestinal villi within the field of view. At least six well-oriented intact villi and their associated crypts (CD) per section were randomly selected and measured for each pig [30].

### 2.4. Intestinal Enzyme Activity Assay

The activity of each enzyme in the intestine was measured using the α-Amylase Assay Kit (mlbio, Shanghai, China), Lipase Assay Kit (Nanjing Jiancheng Bioengineering Institute, Nanjing, China), and β-Galactosidase Assay Kit (mlbio, Shanghai, China), according to the manufacturers’ instructions. The intestinal segments stored at −80 °C were retrieved, and a measured amount was added to distilled water. The mixture was then mechanically homogenized in an ice-water bath. The prepared homogenate was centrifuged at 12,000 *g* for 10 min at 4 °C, and the supernatant was collected for subsequent enzyme activity measurements [31]. The absorbance value was read using a SpectraMax-iD5 microplate reader (Molecular Devices, Sunnyvale, CA, USA) with SoftMax Pro 7.1 software, and the measured value was calculated using a standard curve. This calculated value of enzyme activity was then corrected using the sample protein concentration.

### 2.5. 16S Ribosomal RNA (rRNA) Amplicon Sequencing

Total DNA of microorganisms in the ileum and colon was extracted using the TGuideS96 magnetic bead method soil/feces genomic DNA extraction kit (Tiangen, Beijing, China). The quality and concentration of the extracted DNA were tested using agarose gel electrophoresis and a micronucleic acid concentration analyzer. The V3–V4 regions of bacterial 16S rRNA were amplified using the standard primers 341F (CCTACGGGNGGCWGCAG) and 806R (GGACTACHVGGGTATCTAAT). The constructed library was quantified using Qubit and Q-PCR. Following this, NovaSeq 6000 was used for PE250 sequencing. Raw reads were quality-filtered using Trimmomatic v0.33 with a sliding window of 4 bp and an average quality threshold of Q < 20, and primer sequences were removed using Cutadapt v1.9.1, to obtain high-quality clean reads (mean: 62,500 valid reads per sample; range: 58,300–68,700). Rarefaction curves (based on 97% OTU similarity, QIIME2 2020.6) plateaued for all samples, indicating that sequencing depth was sufficient to characterize the microbial diversity of ileal and colonic contents. To ensure fair comparison and minimize bias caused by uneven sequencing depth, all samples were rarefied to the minimum read count of 58,300 reads prior to α- and β-diversity analyses. Chimeras were removed from rarefied Clean Reads using UCHIME v4.2 to obtain the final Effective Reads data. Operational Taxonomic Units (OTUs) results were obtained by clustering reads at the 97% level using Usearch v10 software. Taxonomic annotation of the feature sequences was performed using a simple Bayesian classifier with SILVA database (release 138) as the reference database, and the composition of each grouped community was determined at the phylum and genus levels. A species abundance table was generated using the QIME2 2020.6 software, and a species distribution histogram was drawn using R language tools. To identify the differences in bacterial communities between adjacent developmental phases, linear discriminant analysis effect size (LEfSe) was employed in this study to screen for differential species. The screening criteria were as follows: the Kruskal–Wallis rank sum test was used for intergroup significance screening with a significance level set at *p* < 0.05; the threshold for the linear discriminant analysis (LDA) effect size was set to LDA score > 2.0; and the Benjamini–Hochberg method was simultaneously adopted for multiple test correction to reduce false positive results [32]. Finally, QIIME2 2020.6 was used to evaluate the alpha diversity index of each group and conduct a beta diversity analysis to obtain the diversity index and Principal Coordinates Analysis (PcoA) results. SPSS 24.0 (International Business Machines Corporation, Armonk, NY, USA) was used to conduct *t*-tests on the species abundance data between groups. Raw Reads were deposited in NCBI’s Serial Read Archive database.

### 2.6. LC-MS Analysis

Samples of the ileal and colonic contents were collected, and each sample was mixed in equal volumes to obtain Quality Control (QC) samples [33,34]. Ground samples (100 mg) were collected in EP tubes, treated with 500 μL of 80% methanol, mixed well, and placed in an ice bath for 5 min. This was followed by centrifugation at 15,000 *g* and 4 °C for 20 min, and the supernatant was aspirated. The supernatant was diluted with water to a methanol concentration of 53%, and centrifuged again at 15,000 *g* and 4 °C for 20 min. This second supernatant was then aspirated for LS-MS analysis, which was performed by Novogene Co., Ltd. (Beijing, China). The detected metabolites were annotated using the KEGG, HMDB, and LIPIDMaps databases, and the data were analyzed using metaX (version 1.5.2) software. Images were drawn using pheatmap (version 1.0.12), ggplot2 (version 3.4.4), and corrplot (version 0.92) packages in R (version 4.3.1).

### 2.7. Statistical Analysis

Raw data were initially organized using Excel 2010 and graphically plotted using GraphPad Prism 9.0 software. Age was included as a fixed effect in the statistical model to adjust for potential age variation within each developmental phase. Statistical analyses were performed using SPSS 24.0. Differences among groups were evaluated by Duncan’s multiple range test. Results are presented as means ± standard error (means ± SE). Differences in alpha diversity (Chao1 and Shannon indices) and genus-level microbial abundance between groups were analyzed using the Wilcoxon rank-sum test, followed by false discovery rate (FDR) correction for multiple testing. Statistical significance was defined as *p* < 0.05, and highly significant as *p* < 0.01.

## 3. Results

### 3.1. Weight Gain and Intestinal Morphological Changes in Wuzhishan Pigs at Different Developmental Phases

**Figure 1:**
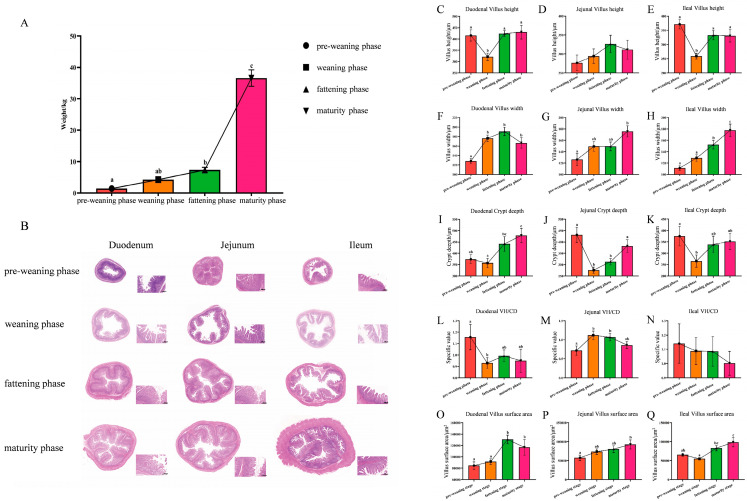
Body weight and intestinal morphological changes in Wuzhishan pigs at different developmental phases. (**A**) Body weights of Wuzhishan pigs at the pre-weaning, weaning, fattening, and maturity phases. (**B**) Sections of duodenum, jejunum, and ileum from the four developmental phases of Wuzhishan pigs. (**C**–**Q**) Morphologic changes in the duodenum, jejunum, and ileum during the four developmental phases of Wuzhishan pigs. (**C**–**E**) Villus height. (**F**–**H**) Villus width. (**I**–**K**) Crypt depth. (**L**–**N**) Villus height-to-crypt depth ratio. (**O**–**Q**) Chorionic surface area. The four developmental phases were defined as follows: pre-weaning phase (7 and 14 days old), weaning phase (35, 38, and 45 days old), fattening phase (70 and 100 days old), and maturity phase (180 and 240 days old). Different letters indicate significant differences (*p* < 0.05). Data are expressed as mean ± SEM; weaning phase, n = 18; other phases, n = 12. Means in a row lacking a common superscript differ (*p* < 0.05).

Body weight increased slowly before the fattening phase. Although body weight was higher in the weaning phase than in the pre-weaning phase, the difference was not significant. Similarly, body weight in the fattening phase was higher than that in the weaning phase, but the difference also did not reach significance (*p* > 0.05) (Figure 1A). The non-significant differences between these early phases may be due to high individual variation and slow weight gain during these developmental phases. In contrast, body weight increased rapidly after the fattening phase, with a significant increase in the mature phase compared with the fattening phase (*p* < 0.05) (Figure 1A). Specifically, body weight increased by an average of 29.3 kg from the fattening phase to the mature phase, accounting for 79.9% of the pre-slaughter weight.

Intestinal morphology is an important indicator of intestinal health [35]. We used hematoxylin and eosin staining to examine the morphology of the duodenum, jejunum, and ileum at different phases of intestinal development. All morphological measurements were performed blindly by independent observers to avoid bias. During the weaning phase, the VH of the duodenum became significantly shorter, the ratio of villus height to crypt depth (VH/CD) decreased significantly, and the VW increased significantly compared to those during the pre-weaning phase. Compared with other phases of development, the pre-weaning phase exhibited the highest VH/CD ratio and shortest VW for the duodenum. During the fattening and maturity phases, the VH, VW, CD, and villi surface area increased with increasing age (*p* < 0.05) (Figure 1C–O). The depth of the jejunum crypt decreased significantly during the weaning phase compared with that during the pre-weaning phase (*p* < 0.05) (Figure 1J). In addition to the depth of the jejunum crypt, other indicators, including jejunum VH, VW, VH/CD ratio, and villi surface area, increased with increasing pig age, among which jejunum VW and villi surface area were the largest during the mature phase (*p* < 0.05) (Figure 1D,G,M,P). Ileal VH and CD were significantly reduced during weaning compared to those during the pre-weaning phase (*p* < 0.05) (Figure 1E,K). Overall, the ileal VH/CD ratio gradually decreased with increasing age, with it being the lowest during the mature phase. In contrast, ileal VW and villi surface area increased with an increase in age, peaking during the mature phase (*p* < 0.05) (Figure 1B,H,N,Q). These results highlight the poor evaluation of intestinal morphology in Wuzhishan pigs during the weaning phase, underscoring the need to focus on their intestinal health during this phase.

### 3.2. Changes in Intestinal Enzyme Activities in Wuzhishan Pigs at Different Developmental Phases

**Figure 2:**
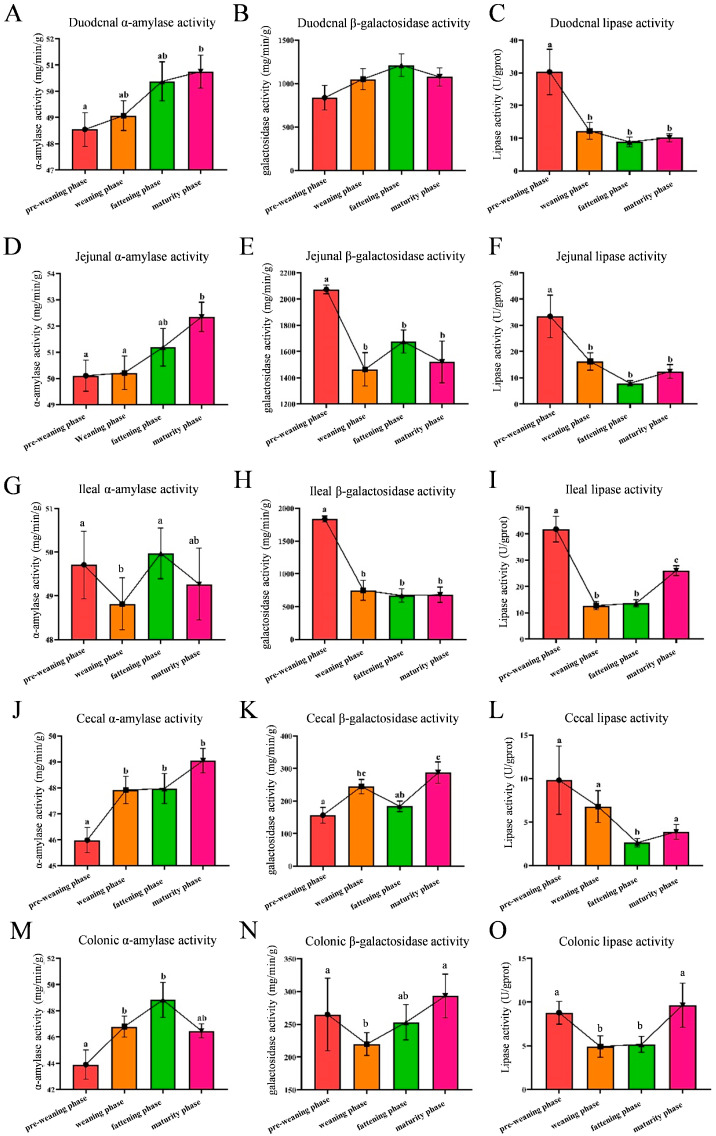
Changes in intestinal digestive enzyme activities at different developmental phases in Wuzhishan pigs. (**A**,**D**,**G**,**J**,**M**) a-Amylase activity of the duodenum, jejunum, ileum, cecum, and colon. (**B**,**E**,**H**,**K**,**N**) β-galactosidase activity in the intestines. (**C**,**F**,**I**,**L**,**O**) Lipase activity in the intestines. Data are expressed as mean ± SEM; weaning phase, n = 18; other phases, n = 12. Means in a row lacking a common superscript differ (*p* < 0.05).

Intestinal enzyme activity is an important component of intestinal digestive function and is closely associated with nutrient digestion and absorption, which in turn supports growth and development in animals. α-Amylase activity in the duodenum, jejunum, caecum, and colon increased with the age of pigs, and reached its peak during the mature phase (*p* < 0.05) (Figure 2A,D,J,M). However, the α-amylase activity in the ileum decreased significantly during the mature phase compared with that during the pre-weaning phase (*p* < 0.05) (Figure 2G).

β-galactosidase activity in the duodenum did not change significantly during the four different developmental phases. In the jejunum and ileum, it was the highest during the pre-weaning phase, but significantly lower during the other three phases (*p* < 0.05) (Figure 2E,H). In the caecum, β-galactosidase activity was significantly increased during the weaning phase compared with that during the pre-weaning phase, and the enzyme activity reached its peak during maturity (*p* < 0.05) (Figure 2K). Unlike the caecum, β-galactosidase activity in the colon decreased significantly during the weaning phase compared with that during the pre-weaning phase (*p* < 0.05) (Figure 2N). Overall, the β-galactosidase activity in the large intestine of Wuzhishan pigs was lower than that in the small intestine.

Lipase activity in the duodenum, jejunum, and ileum was the highest during the pre-weaning phase, but significantly reduced during the other three phases (*p* < 0.05) (Figure 2C–I). In the colon, lipase activity decreased during the weaning and fattening phases (*p* < 0.05) (Figure 2L). In the caecum, it decreased significantly during the fattening phase (*p* < 0.05) (Figure 2O). Overall, lipase activity in the large intestine of Wuzhishan pigs was generally low at <10 U/g prot.

### 3.3. Analysis of Differences in Intestinal Microbiota of Wuzhishan Pigs at Different Developmental Phases

#### 3.3.1. Microbiota Alpha Diversity in the Intestine

**Figure 3:**
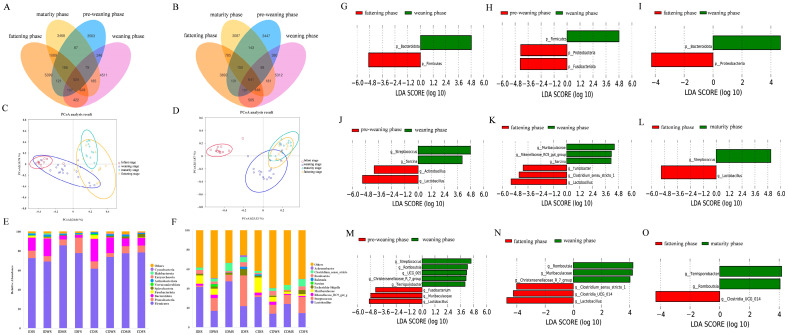
Microbiota composition and differences in the ileum and colon of Wuzhishan pigs at four developmental phases. Venn diagrams of ileum (**A**) and colon (**B**) based on OTU levels. Beta diversity shown in PCoA plots of the ileum (**C**) and colon (**D**) based on OTU levels. Relative abundance of colon and ileum microbiota at phylum (**E**) and genus (**F**) levels. (**G**) Differences in the ileum at the phylum level. (**H**,**I**) Differences in the colon at the phylum level. (**J**–**L**) Differences in the ileum at the genus level. (**M**–**O**) Differences in the colon at the genus level.

To explore the relationship between intestinal microorganisms and the health and development of the Wuzhishan pig intestinal tract, we performed microbial 16S rRNA sequencing analysis of the ileum and colon contents at different developmental phases. As indicated in Figure 3A,B, a Venn diagram was used to reveal the common and unique OTUs of the four different development phases of the Wuzhishan pig. In the ileum, 3447 unique OTUs were identified during the pre-weaning phase, 5312 during the weaning phase, 3893 during the fattening phase, 3087 during the mature phase, and 641 common OTUs during all phases. In the colon, 3563 distinct OTUs were revealed during the pre-weaning phase, 4511 during the weaning phase, 5399 during the fattening phase, 3468 during the mature phase, and 524 OTUs common to all four phases.

In this study, the alpha diversity of the microbiota in the ileum and colon of Wuzhishan pigs at different developmental phases was tested. The results showed no significant difference in the richness of microorganisms in the ileum and colon contents between the pre-weaning and weaning phases. Compared to the weaning phase, the fattening phase showed a significant decrease in the Simpson and Shannon indices of the ileum and a significant increase in the Chao1 index of the colon (*p* < 0.05) (Table 5). Compared to the fattening phase, the maturation phase showed a significant decrease in the Chao1 index of the colon (*p* < 0.05) (Table 5). Furthermore, the analysis of inter-group differences in beta diversity presented in the PCoA showed that the diversity of microorganisms in the ileum and colon contents at different developmental phases was significantly different (*p* < 0.05) (Figure 3C,D). Notably, the microbial diversity of the ileal and colonic contents of Wuzhishan pigs exhibited a significant divergence during the weaning phase. This phenomenon could be attributed to the inherent physiological and functional differences between the ileum (the core segment for nutrient absorption) and the colon (the primary site for microbial fermentation), which were further amplified during the nutritional transition from breast milk to solid feed in the weaning phase. Collectively, these results indicate that the weaning phase serves as a critical node for the succession of the gut microbial community in Wuzhishan pigs, reflecting the community characteristics of local pig breeds in which gut microbes and hosts synergistically adapt to weaning stress.

#### 3.3.2. Analysis of Differences in Intestinal Microorganisms

This study compared and analyzed the intestinal microbiota of Wuzhishan pigs at different developmental phases. The stacking plot shows the horizontal distribution of microbial phyla. *Firmicutes*, *Proteobacteria*, and *Bacteroidota* were the dominant phyla in the ileum and colon contents, while *Lactobacillus*, *Streptococcus*, and *Muribaculaceae* were the dominant genera (Figure 3E,F).

To further compare the different microbial classifications in the intestinal contents at different developmental phases, the different bacterial communities between adjacent developmental phases were compared using LEfSe analysis. In the ileum, relative abundances of *Actinomycetes* and *Lactobacillus* were observed to be significantly higher during the pre-weaning phase, while Streptococcus and Sarcina were significantly enriched during the weaning phase (*p* < 0.05) (Figure 3J). *Bacteroidota*, *Muribaculaceae*, *Rikenellaceae_RC9_gut_group*, and *Sarcina* were significantly enriched during the weaning phase, while *Firmicutes*, *Clostridium_sensu_stricto_1*, *Turicibacter*, and *Lactobacillus* were significantly enriched during the fattening phase (*p* < 0.05) (Figure 3G,K). While *Lactobacillus* was significantly enriched during the fattening phase, *Streptococcus* was significantly enriched during the mature phase (*p* < 0.05) (Figure 3L). In the colon, the relative abundance of *Proteobacteria*, *Fusobacteria*, *Fusobacterium*, *Muribaculaceae*, and *Lactobacillus* during the pre-weaning phase increased compared with that of the weaning phase. Meanwhile, the relative abundance of *Firmicutes*, *Streptococci*, *Romboutsia*, and *Terrisporobacter* increased during the weaning phase (*p* < 0.05) (Figure 3H,M). The relative abundance of *Bacteroidota*, *Romboutsia*, *Muribaculaceae*, and *Christensenellaceae_R-7_group* increased during the weaning phase, while that of *Proteobacteria*, *Clostridium_sensu_stricto_1*, *Clostridia_UCG-014*, and *Lactobacillus* increased during the fattening phase (*p* < 0.05) (Figure 3I,N). The relative abundance of *Clostridia_UCG-014* increased during the fattening phase, while that of *Terrrisporobacter* and *Romboutsia* increased during the maturity phase (*p* < 0.05) (Figure 3O).

### 3.4. Metabolomic Analysis of Intestinal Metabolites in Wuzhishan Pigs at Different Developmental Phases

#### 3.4.1. Identification of Intestinal Metabolites

**Figure 4:**
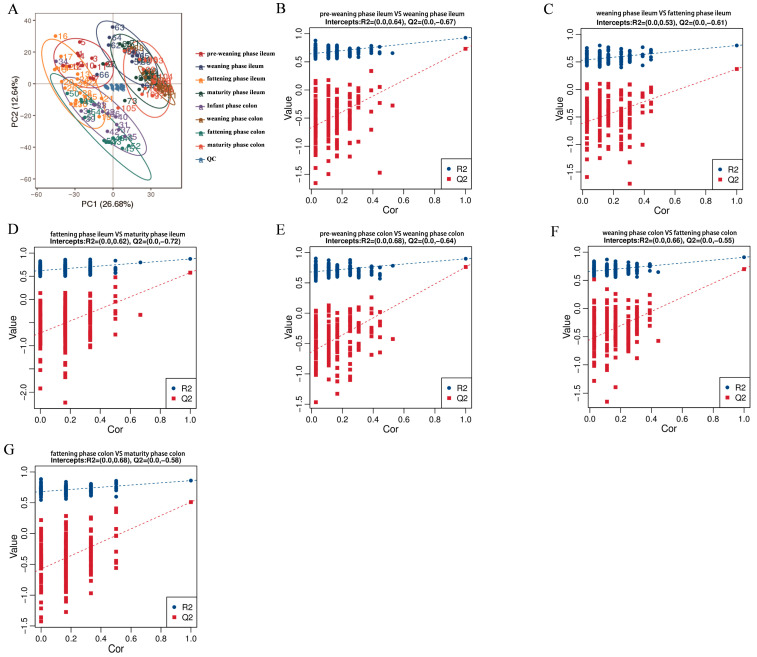
Results of QC-PCA and PLS-DA analysis of intestinal metabolites. (**A**) QC-PCA plot of intestinal metabolites in Wuzhishan pigs. PLS-DA results of two-by-two comparisons between adjacent developmental phases of Wuzhishan pigs are plotted (pre-weaning phase versus weaning phase, weaning phase versus fattening phase, fattening phase versus maturity phase). (**B**–**D**) PLS-DA analysis of porcine ileal contents. (**E**–**G**) PLS-DA analysis of porcine colonic contents.

In the present study, untargeted metabolomics was employed to systematically characterize the dynamic changes in metabolite profiles of ileal and colonic contents in Wuzhishan pigs across different developmental phases. Combined with metabolite functional annotation, phase-specific signature metabolites were identified, thereby providing a theoretical basis for elucidating the metabolic regulatory mechanisms underlying intestinal development in this local pig breed. A total of 2108 metabolites were detected in this experiment. After excluding 782 unclassified compounds, the remaining 1326 metabolites were primarily categorized into 9 major classes, including lipids and lipid-like molecules (523 species, accounting for 39.44%), organic acids and their derivatives (171 species, 19.31%), and organic heterocyclic compounds (167 species, 12.59%). Among these categories, lipids and lipid-like molecules represented the most abundant class, which aligns well with the high-fiber diet tolerance of Wuzhishan pigs. Lipid metabolites are involved in intestinal mucosal barrier formation and energy metabolism, thereby improving the host’s adaptability to high-fiber diets. In addition, quality control principal component analysis (QC-PCA) and correlation analysis revealed that the QC samples exhibited a tight distribution pattern (Figure 4). The relative standard deviations (RSD%) of metabolites in QC samples were well controlled, and low missingness and drift correction were applied during data preprocessing. Results of partial least squares discriminant analysis (PLS-DA) for differential metabolites demonstrated that the model fitting degree (R2) of pairwise comparisons between adjacent developmental phases was consistently higher than the predictive ability (Q2). Additionally, the intercept of the Q2 regression line with the *Y*-axis was less than 0, confirming the absence of overfitting and verifying the reliable predictive power of the model.

#### 3.4.2. Statistical Analysis of Differential Metabolites

**Figure 5:**
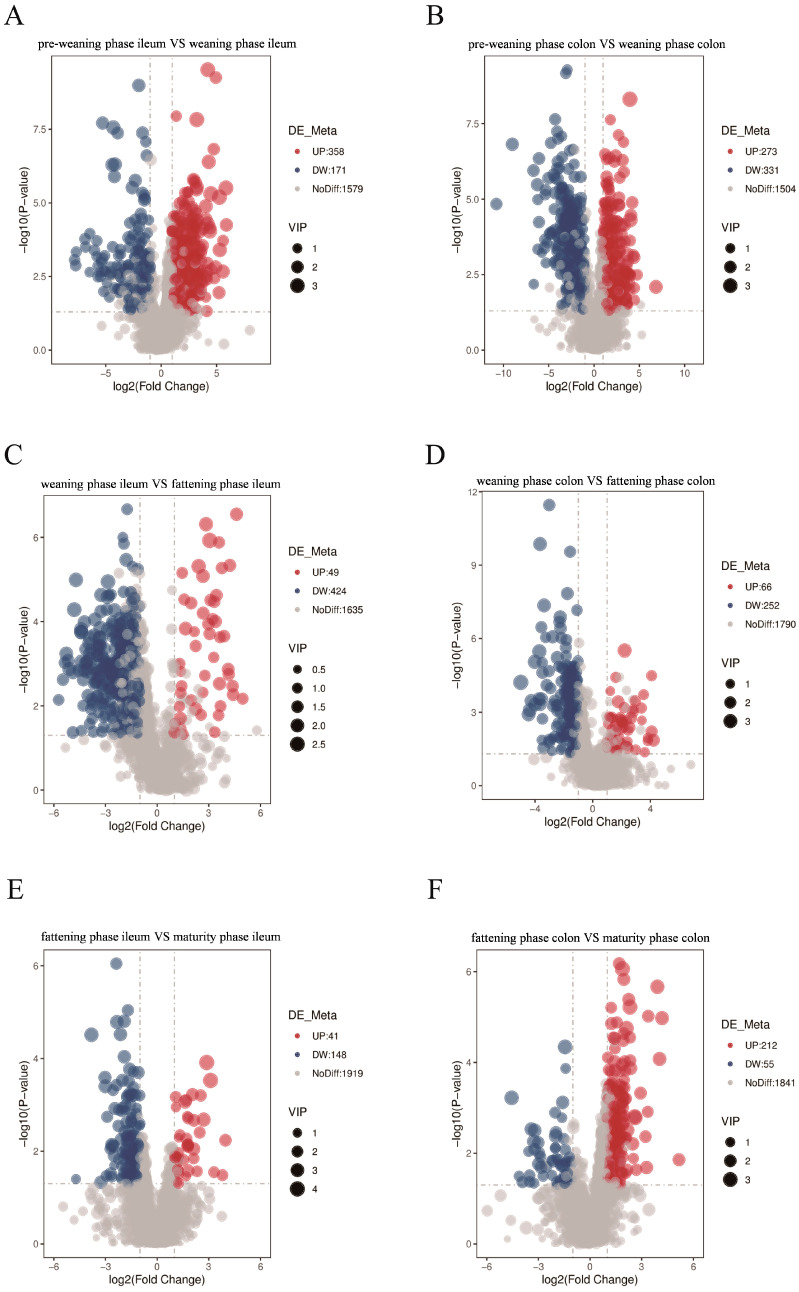
Volcanograms of differential metabolites at the four intestinal development phases in Wuzhishan pigs. Pre-weaning phase versus weaning phase, weaning phase versus fattening phase, fattening phase versus maturity phase. (**A**,**C**,**E**) Volcanograms of differential metabolites of porcine ileal contents. (**B**,**D**,**F**) Volcanograms of differential metabolites of porcine colon contents.

**Figure 6:**
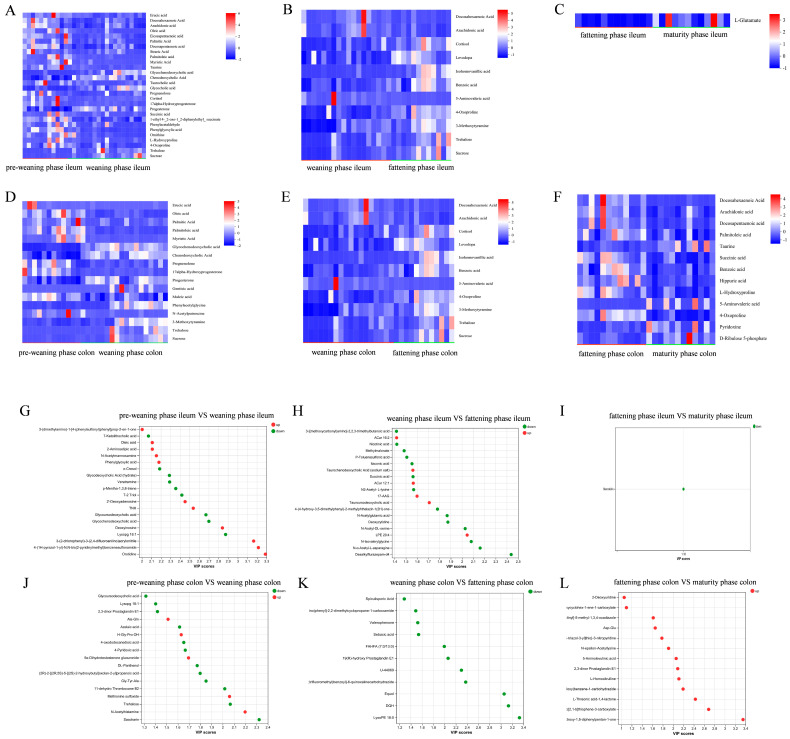
Heatmaps and VIP plots were drawn based on the OPLS-DA model. (**A**–**F**) Metabolite heatmaps. Red indicates a high expression level of the metabolite in the sample, while blue indicates a low expression level. (**G**–**L**) Metabolite VIP plots (VIP > 1). The higher the VIP score, the greater the contribution of the metabolite to the separation of groups.

Statistical analysis of differential metabolites (*p* < 0.05) revealed that the differential patterns of metabolites in the ileum and colon exhibited distinct phase-specificity and segment-specificity. In the ileum, the number of differential metabolites between the pre-weaning and weaning phases was the highest (529 species). A total of 358 metabolites were upregulated in the weaning phase, predominantly lipids and organic acids. Between the weaning and fattening phases in the ileum, 473 differential metabolites were identified, with 424 species showing downregulation in the fattening phase. Only 189 differential metabolites were detected between the fattening and mature phases in the ileum, and Stercobilin (downregulated, VIP = 1.18) was the sole signature metabolite with VIP > 1 (Figure 5). In the colon, 604 differential metabolites were found between the pre-weaning and weaning phases. Among these, 331 species were downregulated in the weaning phase, including carbohydrates such as Saccharin and Trehalose, while upregulated metabolites were dominated by nitrogen-containing compounds (e.g., N-Acetylhistamine). Between the weaning and fattening phases in the colon, 318 differential metabolites were identified, with 252 downregulated metabolites in the fattening phase primarily consisting of Lipids such as LysoPE 18:0. A total of 267 differential metabolites were detected between the fattening and mature phases in the colon, and 212 upregulated metabolites in the mature phase were enriched in antioxidant metabolites including L-Threonic acid-1,4-lactone (Figure 5). To identify key regulatory metabolites, orthogonal partial least squares discriminant analysis (OPLS-DA) was performed to screen for metabolites with VIP > 1 and *p* < 0.05 (Figure 6). The results demonstrated marked functional differentiation between the shared and phase-specific metabolites across different phases. Shared signature metabolites were characterized by the enrichment of lipids and organic acids among differential metabolites during the pre-weaning-weaning transition in both the ileum and colon. Phase-specific signature metabolites included: in the ileum, sedative-like metabolites such as Desalkylflurazepam-d4 were specifically downregulated during the fattening phase; in the colon, anti-inflammatory metabolites, including 3-hydroxy-1,5-diphenylpentan-1-one, were specifically enriched in the mature phase. These shared and specific metabolites can serve as core biomarkers for assessing the intestinal functional status of Wuzhishan pigs at different developmental phases.

#### 3.4.3. KEGG Enrichment Analysis of Differential Metabolites

**Figure 7:**
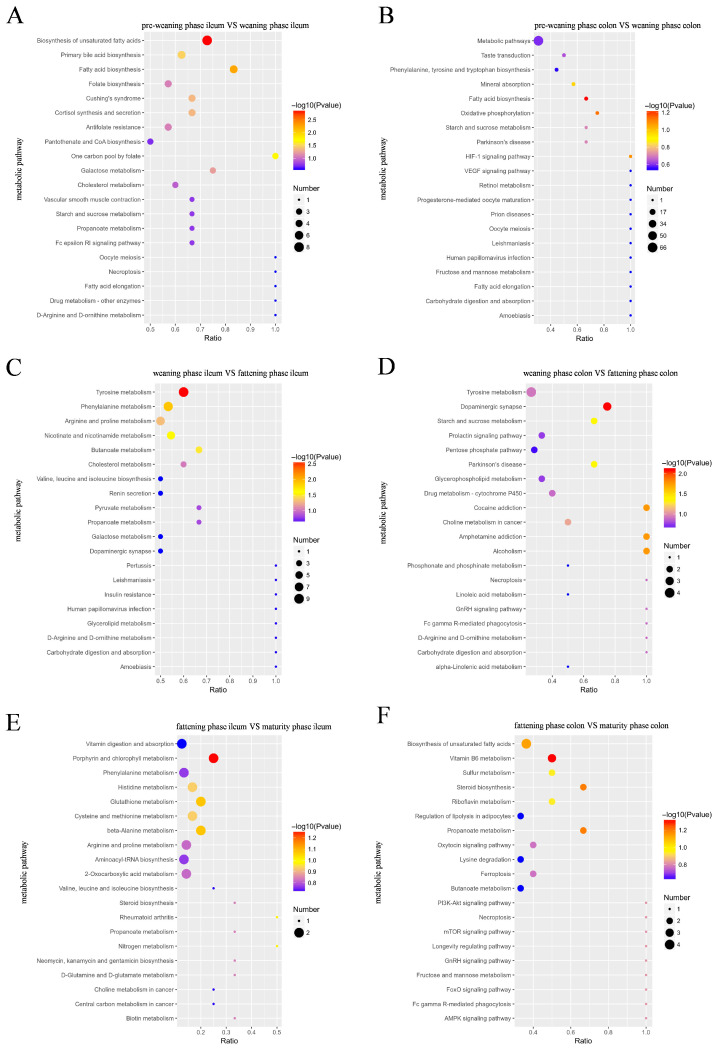
KEGG differential metabolic pathway bubble diagrams of intestinal metabolites at different developmental phases in Wuzhishan pigs. (**A**,**C**,**E**) Bubble plots of KEGG differential metabolic pathways of metabolites from porcine ileum. (**B**,**D**,**F**) Bubble plots of KEGG differential metabolic pathways of metabolites from porcine colon.

The differential metabolites were assigned to the KEGG database for annotation, and a total of 15 KEGG secondary pathways were identified. Consistent with the metabolomic experimental protocol, KEGG pathway enrichment analysis was performed using metaX software, with the hypergeometric test as the enrichment method, all annotated metabolites (annotated via the KEGG, HMDB, and LIPIDMaps databases) as the background set, and the Benjamini–Hochberg method applied for multiple-testing correction to control false positive results. Differential metabolites in the ileal contents at the pre-weaning and weaning phases were mainly enriched in the unsaturated fatty acid biosynthesis, fatty acid biosynthesis, primary bile acid biosynthesis, and cortisol synthesis and secretion metabolic pathways (Figure 7A). The ileal differential metabolites at the weaning and fattening phases were mainly enriched in the tyrosine, phenylalanine, nicotinic acid, nicotinamide, butyric acid, arginine, and proline metabolic pathways (Figure 7C). In addition, the colonic differential metabolites at the weaning and fattening phases were significantly enriched in starch and sucrose metabolic pathways (Figure 7D). Vitamin B6 metabolic pathways were significantly enriched in colonic differential metabolites during the fattening and maturation phases (Figure 7F).

### 3.5. Correlation Analysis Between Microbiota and Metabolites

**Figure 8:**
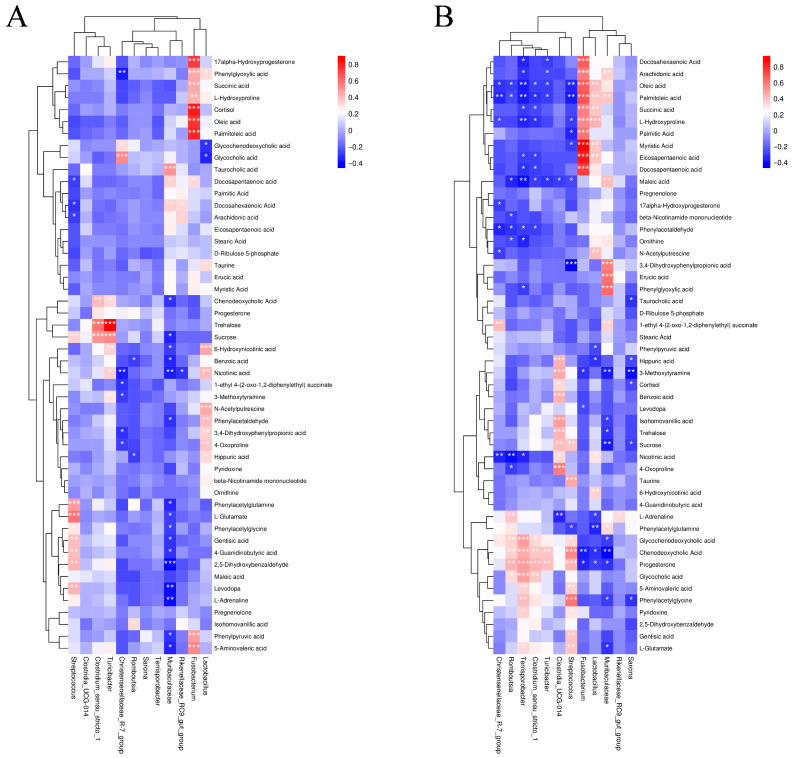
Heat map of correlation between differential microbiota and metabolites in the intestinal contents of Wuzhishan pigs at different developmental phases. (**A**) Spearman’s correlation heatmap of differential microbiota and differential metabolites in the colonic contents. (**B**) Spearman’s correlation heatmap of differential microbiota and differential metabolites in the ileum. The legend shows the magnitude of Spearman’s correlation coefficient; red indicates a positive correlation and blue indicates a negative correlation. * *p* < 0.05, ** *p* < 0.01, *** *p* < 0.001, indicating significant correlations.

Spearman’s rank correlation coefficient and significance tests revealed a correlation between different microbiota and metabolites in the colon and ileum at different phases of development. FDR correction was applied for multiple testing to control false positives. Heat map results showed that the abundance of *Fusobacteria*, *Lactobacillus*, and *Muribaculaceae* in the colon was positively correlated with the concentration of metabolites that affect the unsaturated fatty acid biosynthesis pathway. These metabolites included docosahexaenoic acid, arachidonic acid, oleic acid, eicosapentaenoic acid, palmitic acid, docosapentaenoic acid, and erucic acid. On the other hand, the abundance of *Christensenellaceae_R-7_group*, *Clostridium_sensu_stricto_1*, *Terrisporobacter*, and *Turicibacter* was negatively correlated with the concentration of metabolites such as oleic acid, palmitoleic acid, eicosapentaenoic acid, docosapentaenoic acid, docosahexaenoic acid, arachidonic acid, and erucic acid (*p* < 0.05). The abundance of *Fusobacteria*, *Lactobacillus*, and *Muribaculaceae* was positively correlated with metabolites that affect the fatty acid biosynthesis pathway, including palmitoleic acid, myristic acid, and oleic acid. Of note, the association between Fusobacteria and lipid metabolites does not confirm a beneficial role of this phylum in lipid metabolism, and the exact function of this genus remains to be further studied. Meanwhile, the abundance of *Romboutsia* and *Streptococcus* was inversely correlated with metabolites such as oleic acid, palmitoleic acid, palmitic acid, and myristic acid (*p* < 0.05). The abundance of *Clostriumsensustricto1*, *Rombsia*, *Streptococcus*, and *Turicibacter* was positively correlated with the concentration of metabolites that affect the primary bile acid biosynthesis pathway, including glycochenodeoxycholic acid, glycocholic acid, chenodeoxycholic acid, and taurine. Of note, genus-level 16S rRNA sequencing alone cannot clearly confirm the bile acid transformation capacity of the related bacteria, and further experimental verification is required. The abundance of *Muribaculaceae* was negatively correlated with the concentration of glycochenodeoxycholic acid and chenodeoxycholic acid (*p* < 0.05). In addition, the abundance of *Clostridia_UCG-014* was positively correlated with the concentration of trehalose and sucrose in the starch and sucrose metabolic pathways (*p* < 0.05). While the abundance of *Lactobacillus* was inversely correlated with the concentration of phenylpyruvic acid, hippuric acid, and phenylacetylglutamine in the phenylalanine metabolic pathway (*p* < 0.05), it was positively correlated with the concentration of ornithine, L-hydroxyproline, and N-acetylputresine in the arginine and proline metabolic pathways (*p* < 0.05). *Muribaculaceae* was positively correlated with five metabolites of the tyrosine metabolic pathway, including 1-Ethyl 4-(2-oxo-1,2-diphenylethyl) succinate, 3,4-Dihydroxyphenylpropionic acid, maleic acid, phenylacetylglutamine, and phenylglyoxylic acid, but inversely correlated with homovanillic acid and phenylacetylglycine (*p* < 0.05). The abundance of *Terrisporobacterium* was negatively correlated with the concentration of succinic and maleic acids (*p* < 0.05), which may affect the metabolism of butyrate (Figure 8).

In the ileum, *Fusobacterium*, *Muribaculaceae*, *Rikenellace_RC9_gut_group*, and *Streptococcus* were positively correlated with the concentration of metabolites that affected the unsaturated fatty acid biosynthesis pathway. These included oleic acid, docosapentaenoic acid, docosahexaenoic acid, and arachidonic acid (*p* < 0.05). *Lactobacillus* and *Streptococcus* abundance was positively correlated with the concentration of metabolites in the tyrosine metabolic pathway, such as 3,4-Dihydroxyphenylpropionic acid, hippuric acid, phenylacetylglycine, and phenylacetylglutamine. Meanwhile, the abundance of *Christensenellaceae_R-7_group*, *Muribaculaceae*, and *Romboutsia* was negatively correlated with the concentration of metabolites in the tyrosine metabolic pathway. These included 3,4-Dihydroxyphenylpropionic acid, 1-Ethyl 4-(2-oxo-1,2-diphenylethyl) succinate, benzoic acid, phenylpyruvic acid, phenylacetaldehyde, and hippuric acid (*p* < 0.05). *Fusobacteria* and *Lactobacillus* abundance was positively correlated with the concentration of metabolites in the phenylalanine metabolic pathway, such as phenylpyruvic acid, phenylglyoxylic acid, and phenylacetaldehyde. However, *Muribaculaceae* and *Streptococcus* abundance was negatively correlated with the concentration of metabolites in the phenylalanine metabolic pathway, including levodopa, L-adrenaline, gentisic acid, and 2,5-Dihydroxybenzaldehyde (*p* < 0.05). In addition, *Clostridiumsensustricto1* and *Turicibacter* abundance was positively correlated with the concentration of Trehalose and Sucrose (*p* < 0.05), suggesting that they may be involved in starch and sucrose metabolic pathways (Figure 8).

## 4. Discussion

As one of the six small pig breeds in China, the Wuzhishan pig has excellent traits such as early maturity, high fiber tolerance, heat tolerance, and good meat quality [36]; however, its slow growth and small body size have restricted its development and utilization. This is related to its slow-growing, small-body-size genotype. Wuzhishan pigs inherently have low nutrient requirements, which is why they are able to tolerate roughage. Compared with modern commercial pig genotypes, Wuzhishan pigs possess a superior capacity for fiber utilization and higher fiber digestibility, which underlies their characteristic roughage tolerance. This advantage is closely associated with their unique intestinal microbiota and genetic background related to carbohydrate metabolism. Such efficient fiber utilization allows them to adapt better to low-energy, high-fiber feeding systems than modern improved breeds. The body weight results of Wuzhishan pigs from the pre-weaning phase to maturity in the present study further confirm this characteristic. Rapid body weight gain did not appear until the fattening phase, and the average body weight at maturity was 36.59 kg. The intestinal tract is the largest digestive organ and is crucial for the nutrient absorption, growth, and development of piglets. Therefore, the present study elucidated the morphology, digestive enzyme activity, microbiome, and metabolome of the intestinal tract of Wuzhishan pigs at different developmental phases. These results indicate that intestinal morphology is less developed in weaning-phase Wuzhishan pigs, and improving intestinal health during this phase may benefit their growth performance. The VH/CD ratio, an indicator of intestinal digestive and absorptive capacity [37], decreased with age in the duodenum and ileum but not in the jejunum, suggesting that attention should be paid to the underlying mechanisms contributing to this change. During the weaning phase, the VH of the duodenum and ileum were significantly shorter, whereas the VW was significantly increased. This seemingly contradictory change was not caused by villus edema or section orientation bias, but likely represents an adaptive and compensatory response to weaning stress, in which the intestine increases the villus width to expand the effective absorptive surface area, thereby compensating for the reduction in nutrient absorption capacity caused by the shortened villus height.

In addition to intestinal morphology, digestive enzyme activity is a key factor affecting the digestion and absorption of nutrients. Therefore, we tested the activities of digestive enzymes such as α-amylase, β-galactosidase, and lipase at different developmental phases. α-Amylase breaks the α-1,4 glycosidic bond of starch and related polysaccharide molecules, hydrolyses them into substances such as maltose and dextrins, and facilitates the absorption of starch in the body [38]. Our results showed that the α-amylase activity increased with increasing age and was highest at the maturation phase in the duodenum, jejunum, caecum, and colon; however, in the ileum, α-amylase activity was significantly decreased at the maturity phase. This may be attributed to segment-specific intestinal functional changes during development in Wuzhishan pigs. β-Galactosidase is mainly responsible for catalyzing lactose hydrolysis and transglycosidation [39]. The study found that the β-galactosidase activity in the jejunum and ileum of Wuzhishan pigs was higher during the pre-weaning phase than during the other growth phases. β-Galactosidase activity decreased significantly during the weaning phase in the colon. This may be related to a decrease in the activity of enzymes secreted by the brush border of the villi due to feed changes and damage to the intestinal morphology during weaning. In pig intestines, lipase breaks down fat into glycerol and fatty acids, allowing the fat to be fully digested and absorbed by the body [40]. Our research results showed that the lipase activity in the large and small intestines of Wuzhishan pigs was the highest during the pre-weaning phase, with higher activity in the small intestine than in the large intestine. This may be related to the greater fat content in pig milk [41] and the high demand for lipolysis during the pre-weaning phase. To sum up, we hypothesize that the slow growth disadvantage of Wuzhishan pigs could potentially be alleviated by increasing α-amylase and β-galactosidase activity during the weaning phase, as well as enhancing lipase activity during the weaning, fattening, and maturation phases, which may promote efficient nutrient digestion and absorption.

Intestinal microbiota is a key regulator of nutrient metabolism, which can affect the host’s nutrient digestion, absorption, growth, and development through metabolic activities, and its structure and composition are also crucial for maintaining intestinal homeostasis and disease resistance [42]. We therefore analyzed the successional characteristics of ileal and colonic microbiota in Wuzhishan pigs across pre-weaning, weaning, fattening, and maturity phases. In this study, the number of differential bacterial species among groups was relatively small. This may be explained by two factors: first, as a roughage-tolerant native breed, Wuzhishan pigs have a stable intestinal microbiota shaped by long-term co-evolution, with responses to external stress dominated by fine adjustments in abundance rather than drastic shifts in dominant species; second, the main difference between ileum and colon lies in the abundance changes of functional bacteria rather than large-scale compositional turnover. Alpha-diversity results showed that the richness and diversity of microbial communities at different developmental phases differed with significant phase-specificity. This was shown by the significantly lower Simpson and Shannon indices of the ileum during the fattening phase than those during the weaning phase (*p* < 0.05). However, the Chao1 indices of the colon were significantly higher than those during the weaning phase (*p* < 0.05), reaching a peak in the fattening phase, while those during the maturation phase were significantly lower than those during the fattening phase (*p* < 0.05). The digestive system of pigs at the mature phase, including intestinal villus structure and digestive enzyme profiles, has fully developed and stabilized, with immune function reaching its peak, thus forming a strict intestinal microecological “selective barrier”. This barrier only allows the long-term colonization of dominant microbiota highly compatible with the host’s metabolic needs, while the auxiliary microbiota rapidly colonized during the weaning and fattening phases may be inhibited or eliminated due to their inability to adapt to the physiological environment of the digestive system in mature pigs (such as pH value, digestive enzyme profile, and immune factor level), directly leading to a decrease in microbial diversity. This is consistent with the conclusion reported by Lee et al. [43] that “during the stable developmental phase of healthy hosts, with the gradual improvement of intestinal physiological homeostasis, the selective colonization effect of the intestine on the microbiota is enhanced, and the specificity of microbiota colonization is significantly increased, which may lead to a decreasing trend in species richness compared with the phase when homeostasis is not established”, further verifying the rationality of the changes in intestinal microbiota diversity observed in this study. Healthy gut microbiota is usually composed of four main phyla: Firmicutes, Bacteroidetes, Actinobacteria, and Proteobacteria [44]. In this study, we observed that the proportion of Firmicutes in the intestinal tract of Wuzhishan pigs was proportional to age, whereas the proportion of Bacteroides showed the opposite trend. The dynamic adjustment of the ratio between them may be the key microecological driving factor for the rapid weight gain of Wuzhishan pigs in the later phases of development. Studies have shown that changes in the ratio of Firmicutes to Bacteroides affect the digestion and absorption of fat in pigs. An increase in the proportion of Bacteroides in the intestinal microbiota correlates with weight loss, whereas an increase in the proportion of Firmicutes correlates with lipid accumulation [45], which is consistent with the enrichment of lipid metabolites in the mature phase in the previous metabolomics results, explaining the growth characteristics of Wuzhishan pigs from the perspective of microbiota-metabolism interaction. In addition, *Streptococcus*, *Romboutsia*, and *Terrisporobacter* often cause an imbalance in the intestinal microbiota, which is closely related to intestinal inflammation [46]. This study found that at the genus level, *Streptococcus*, *Romboutsia*, and *Terrisporobacter* increased significantly during the weaning phase of Wuzhishan pigs (*p* < 0.05). With an increase in age, *Streptococcus* became the dominant bacterial genus during the mature phase, suggesting that the weaning phase of Wuzhishan pigs, a stressful environment (such as feed conversion and intestinal structure remodeling), has a profound impact on intestinal damage and provides an opportunity for the colonization of harmful bacteria. This also explains the fluctuation of anti-inflammatory metabolites in the metabolomics during the weaning phase. Notably, although the weaning phase of Wuzhishan pigs was dominated by some pathogenic bacteria, the beneficial bacteria *Muribaculaceae*, *Rikenellaceae_RC9_gut_group*, and *Christensenellaceae_R-7_group* were significantly enriched in the ileum and colon (*p* < 0.05), which play a role in maintaining intestinal health and preventing inflammation and infections by regulating intestinal short-chain fatty acid metabolism and inhibiting the expression of pro-inflammatory factors [46]. The enrichment of these taxa during weaning may be potentially associated with the resistance to weaning stress and the maintenance of intestinal function in Wuzhishan pigs. We therefore speculate that maintaining intestinal microbiota stability and promoting beneficial bacteria during weaning may help alleviate the slow growth characteristic of Wuzhishan pigs.

The intestinal microbiota maintains a steady state in the intestine and produces bioactive metabolites, which can serve as messengers of the intestinal microbiota. They directly or indirectly regulate multiple physiological processes of the host, including immunity, inflammatory responses, and intestinal barrier function, as well as metabolic pathways [47]. In this study, we identified and screened the changes in metabolites in the intestinal contents of Wuzhishan pigs at different phases of development through non-targeted metabolomics. We performed functional annotations and association analyses of these metabolites and clarified their physiological functions in metabolic pathways, as well as their correlations with differential intestinal microbiota. Metabolomic analysis showed that the lipid metabolism of Wuzhishan pigs during the weaning phase was significantly lower than that during the pre-weaning phase (*p* < 0.05), which was reflected by a decrease in the concentration of metabolites related to lipid metabolism, such as arachidonic acid, erucic acid, and docosahexaenoic acid. Correlation analysis revealed that the abundance of *Fusobacterium*, *Lactobacillus*, and *Muribaculaceae*, which were positively correlated with the concentration of metabolites in the lipid synthesis pathway, decreased during the weaning phase (*p* < 0.05). These results suggest that the weakened lipid digestion and slow weight gain during the weaning phase may be associated with the interaction between intestinal microbiota and lipid metabolism, reflecting the synergistic effect of microbiota-metabolite interactions on lipid digestion and absorption in weaned piglets. Notably, our metabolomic results showed that 358 metabolites were upregulated in the ileum during the weaning phase, predominantly lipids and organic acids, which is presumably associated with the remodeling of intestinal energy metabolism in response to weaning stress. Additionally, both the ileum and colon showed enrichment of lipids and organic acids among differential metabolites during the pre-weaning-weaning transition, indicating that, despite functional divergence between the two intestinal segments, there are common metabolic regulatory pathways in response to weaning stress. In the colon, upregulated metabolites during the pre-weaning-weaning transition were dominated by nitrogen-containing compounds (e.g., N-Acetylhistamine), which is closely linked to the initiation of microbial fermentation function in the colon—consistent with the synergistic interaction between intestinal microbiota and metabolites observed in this study. Therefore, appropriate fat supplementation during the suckling-to-weaning transition can enhance immunity, alleviate inflammation, and improve body and intestinal health in Wuzhishan piglets, thereby minimizing the negative impacts of weaning stress on their growth and ensuring normal growth performance. Compared to the weaning phase, the fattening phase showed increased amino acid metabolism. This was manifested by an increased content of amino acids such as ornithine, L-glutamate, and L-hydroxyproline in the intestine, and metabolites related to muscle development such as succinic acid and nicotinic acid (*p* < 0.05) [48,49,50]. Correlation analysis revealed that *Lactobacillus* positively correlated with the concentration of metabolites in the amino acid metabolic pathway, and the abundance of this bacterium increased significantly during the fattening phase (*p* < 0.05), indicating that *Lactobacillus* may promote amino acid synthesis and transformation to provide a sufficient material basis for muscle development.

Wuzhishan pigs, a typical indigenous breed with slow-growing but stress-tolerant genotypes, differ distinctly from modern commercial genotypes selected for rapid growth. The downregulation of sedative-like metabolites (e.g., Desalkylflurazepam-d4) in the ileum during the fattening phase reflects a breed-specific compensatory growth mechanism, which is less prominent in modern genotypes that rely on high-nutrient diets rather than intrinsic metabolic adjustments. The establishment of intestinal metabolic homeostasis (evidenced by reduced ileal differential metabolites and stercobilin as the sole signature metabolite) is a key trait of Wuzhishan pigs’ genotype, in contrast to modern commercial genotypes, which exhibit fluctuating intestinal metabolism due to stress sensitivity. This stability is linked to their unique protein and fat deposition patterns, distinguishing them from modern breeds that exhibit rapid but unstable weight gain. The enrichment of anti-inflammatory and antioxidant metabolites in the colon during the mature phase underpins Wuzhishan pigs’ roughage tolerance, a genetic advantage that has largely diminished in modern genotypes selected for grain-based diets. Notably, Wuzhishan pigs have genotype-specific nutritional requirements that differ from those of modern commercial pigs, which thrive on high-protein diets; targeted supplementation of ornithine and L-glutamate enhances their compensatory weight gain, aligning with their low-nutrient-demand genotype. Collectively, these findings decipher the metabolic mechanisms underlying the unique traits of Wuzhishan pigs, highlighting the value of their genotype for improving stress resistance in commercial breeding—their metabolic stability offers a model for sustainable feeding strategies compared to modern genotypes.

## 5. Conclusions

This study systematically explored the intestinal physiological differences of Wuzhishan pigs across pre-weaning, weaning, fattening, and mature phases from intestinal morphology, digestive enzyme activity, microbiota diversity, and metabolite characteristics, clarifying that their intestinal development has significant phase-specificity related to growth performance and breed advantages. During weaning, intestinal morphological damage, reduced digestive enzyme activity, and opportunistic pathogens (Streptococcus, Romboutsia, Terrrisporobacter) caused slow early growth, with depressed lipid metabolism exacerbating this. In fattening, stable metabolism, enriched Lactobacillus (positively correlated with ornithine, L-glutamate, succinic acid, and nicotinic acid), and enhanced amino acid metabolism promoted growth, related to their roughage tolerance and low nutritional requirements. The mature phase had increased immune-related metabolites (e.g., pyridoxine) in the vitamin B6 pathway. This study clarified microbiota-metabolite dynamic changes, screened functional metabolites, and revealed unique intestinal mechanisms, filling research gaps and providing a theoretical basis for improving growth and application value via nutritional regulation and feeding management.

## Figures and Tables

**Table 1:** Nutrient content of the lactating sow feed (as-fed basis, %).

Item	Content
Crude ash	5.5
Moisture	13.2
Crude fiber	4
Crude protein	16.58
Total phosphorus	0.58
Calcium	0.733
Crude fat	3.3
Total amino acids	13.59
Digestible energy, MJ/kg	18.78

Notes: Amino acid additives: L-lysine 1.04%, DL-methionine 0.14%, L-threonine 0.54%, L-valine 0.72%, L-isoleucine 0.64%, L-leucine 1.23%, L-phenylalanine 0.83%, L-histidine 0.42%, L-arginine 0.83%, aspartate 1.37%, serine 0.66%, glutamate 2.55%, proline 0.85%, glycine 0.60%, alanine 0.72%, tyrosine 0.45%.

**Table 2:** Nutrient content of the weaning piglet diets (as-fed basis, %).

Item	Content
Crude ash	5.9
Moisture	11.8
Crude fiber	3.4
Crude protein	16.95
Total phosphorus	0.52
Calcium	0.798
Crude fat	6.6
Total amino acids	15.24
Digestible energy, MJ/kg	18.82

Notes: Amino acid additives: L-lysine 1.33%, DL-methionine 0.17%, L-threonine 0.73%, L-valine 0.78%, L-isoleucine 0.71%, L-leucine 1.34%, L-phenylalanine 0.88%, L-histidine 0.46%, L-arginine 0.94%, aspartate 1.60%, serine 0.73%, glutamate 2.75%, proline 0.94%, glycine 0.64%, alanine 0.76%, tyrosine 0.48%.

**Table 3:** Nutrient content of the fattening pig diets (as-fed basis, %).

Item	Content
Crude ash	4.6
Moisture	11.8
Crude fiber	4.8
Crude protein	13.17
Total phosphorus	0.5
Calcium	0.786
Crude fat	5.2
Total amino acids	11.18
Digestible energy, MJ/kg	19.03

Notes: Amino acid additives: L-lysine 0.85%, DL-methionine 0.15%, L-threonine 0.50%, L-valine 0.60%, L-isoleucine 0.46%, L-leucine 1.09%, L-phenylalanine 0.68%, L-histidine 0.34%, L-arginine 0.63%, aspartate 0.89%, serine 0.55%, glutamate 2.13%, proline 0.77%, glycine 0.49%, alanine 0.66%, tyrosine 0.39%.

**Table 4:** Nutrient content of the mature pig diets (as-fed basis, %).

Item	Content
Crude ash	6.4
Moisture	12.1
Crude fiber	16.3
Crude protein	9.29
Total phosphorus	0.46
Calcium	0.447
Crude fat	2.3
Total amino acids	7.29
Digestible energy, MJ/kg	18.57

Notes: Amino acid additives: L-lysine 0.36%, DL-methionine 0.07%, L-threonine 0.28%, L-valine 0.43%, L-isoleucine 0.29%, L-leucine 0.60%, L-phenylalanine 0.46%, L-histidine 0.25%, L-arginine 0.47%, aspartate 0.62%, serine 0.37%, glutamate 1.49%, proline 0.51%, glycine 0.40%, alanine 0.44%, tyrosine 0.25%.

**Table 5:** Alpha diversity index.

Items	Pre-Weaning Phase	Weaning Phase	Fattening Phase	Mature Phase
Ileum				
Chao1 index	926.41 ± 54.23	973.96 ± 31.90	1033.17 ± 101.59	748.41 ± 175.12
Simpson index	0.96 ± 0.01 ^a^	0.93 ± 0.02 ^a^	0.86 ± 0.03 ^b^	0.83 ± 0.02 ^b^
Shannon index	6.36 ± 0.21 ^a^	6.74 ± 0.26 ^a^	5.10 ± 0.37 ^b^	4.58 ± 0.44 ^b^
Colon				
Chao1 index	834.92 ± 40.72 ^a^	852.18 ± 53.67 ^a^	1356.59 ± 106.98 ^b^	1010.60 ± 127.35 ^a^
Simpson index	0.95 ± 0.01	0.95 ± 0.01	0.94 ± 0.01	0.94 ± 0.01
Shannon index	5.95 ± 0.13 ^a^	6.67 ± 0.28 ^ab^	6.81 ± 0.28 ^b^	6.27 ± 0.29 ^ab^

Means in a row lacking a common superscript differ (*p* < 0.05). The four developmental phases were defined as follows: pre-weaning phase (7 and 14 days old), weaning phase (35, 38, and 45 days old), fattening phase (70 and 100 days old), and maturity phase (180 and 240 days old). Each value represents the mean ± SEM (weaning phase n = 18 and other phases n = 12).

## Data Availability

Raw sequencing data are available in the NCBI BioProject database (BioProject ID: PRJNA1246071).

## Abbreviations

The following abbreviations are used in this manuscript:

VH	Villus height
VW	Villus width
OTUs	Operational taxonomic units
PcoA	Principal coordinates analysis
QC	Quality control
KEGG	Kyoto Encyclopedia of Genes and Genomes
LefSe	Linear discriminant analysis effect size
VIP	Variable importance in projection
